# Assessment of antinuclear antibodies (ANA): National recommendations on behalf of the Croatian society of medical biochemistry and laboratory medicine

**DOI:** 10.11613/BM.2021.020502

**Published:** 2021-04-15

**Authors:** Andrea Tešija Kuna, Lovorka Đerek, Vedrana Drvar, Ana Kozmar, Katarina Gugo

**Affiliations:** 1 Department of Clinical Chemistry, Sestre Milosrdnice University Hospital Center, Zagreb, Croatia; 2 Clinical Department of Laboratory Diagnostics, University Hospital Dubrava, Zagreb, Croatia; 3 Clinical Department of Laboratory Diagnostics, Clinical Hospital Center Rijeka, Rijeka, Croatia; 4 Department of Laboratory Diagnostics, University Hospital Center Zagreb, Zagreb, Croatia; 5 Department of Medical Laboratory Diagnostics, University Hospital Center Split, Split, Croatia

**Keywords:** antinuclear antibodies, autoimmunity, recommendations, harmonization

## Abstract

Antinuclear antibodies (ANA) represent a family of autoantibodies targeting ubiquitous cellular constituents and are a hallmark of systemic inflammatory autoimmune rheumatic diseases named connective tissue diseases (CTD). The gold standard method for ANA determination is indirect immunofluorescence (IIF) on the human laryngeal epidermoid carcinoma cell line type 2 substrate (HEp-2), but with increasing demand for ANA testing, novel methods eased for automation emerged, which allows testing by staff less experienced in this specific field of laboratory diagnostic. In 2016 The working group (WG) for laboratory diagnostics of autoimmune diseases as part of the Committee for the Scientific Professional Development of the Croatian Society of Medical Biochemistry and Laboratory Medicine (CSMBLM) published the data of a survey regarding general practice in laboratory diagnostics of autoimmune diseases in Croatia. Results indicated high diversity in the performance of autoantibody testing as well as reporting of the results and indicated the need of creating recommendations for the assessment of ANA that would help harmonize diagnostics of systemic autoimmune rheumatic diseases in Croatia. This document encompasses twenty-seven recommendations for ANA testing created concerning indications for ANA testing, preanalytical, analytical, and postanalytical issues, including rational algorithm and quality control assurance. These recommendations are based on the relevant international recommendations and guidelines for the assessment of ANA testing and relevant literature search and should help to harmonize the approach in ANA testing and clarify differences in interpretation of the results obtained using different methods of determination.

## Introduction

Autoantibodies are a hallmark of autoimmunity, of which antinuclear antibodies (ANA) have the historically central role (*1*, *2*). Antinuclear antibodies comprise a diverse group of autoantibodies directed against multiple intra-cellular antigens at various cellular compartments including nuclear constituents (chromatin, nucleoli, and nucleoplasm), components of the nuclear envelope, mitotic spindle apparatus, and cytosol (*1*). The growing number of newly characterized target autoantigens and evidence of their role in certain autoimmune diseases resulted in the continuing broadening of test panels with concomitant evolution of methods and analytical systems in this specific field of laboratory diagnostics. Unfortunately, as a drawback of this trend there is a huge heterogeneity in nomenclature in use, algorithms of ANA testing, analytical methodology, results reporting, and interpretation. The results of the survey launched by the Working group (WG) for laboratory diagnostics of autoimmune diseases of the Croatian Society of Medical Biochemistry and Laboratory Medicine (CSMBLM) confirmed this heterogeneity throughout medical laboratories in Croatia and prompted the creation of the first national recommendations for the assessment of ANA (*3*).

These national recommendations are based on the International recommendations for the assessment of anti-nuclear antibody testing published by the European Autoimmunity Standardization Initiative (EASI) and are directed to pre-analytical issues (including rationale algorithm), analytical issues, and particularly to reporting and interpretation of the results (*1*).

Antinuclear antibodies are a hallmark of the subgroup of systemic inflammatory autoimmune rheumatic diseases (SARD) named connective tissue diseases (CTD) which includes: systemic lupus erythematosus (SLE), primary Sjögren syndrome (SjS), Systemic sclerosis (scleroderma, SSc), idiopathic inflammatory myopathies (IIMs), mixed connective tissue disease (MCTD) and overlap syndromes (*4*–*6*). Antinuclear antibodies represent classification criteria of most CTD while it is a fundamental parameter for diagnosis of autoimmune hepatitis (AIH) as an organ-specific autoimmune disease and validated risk factor for the development of uveitis in patients with juvenile idiopathic arthritis (JIA) (*7*, *8*).

Nomenclature of ANA specificities originates from the biochemical properties of targeted antigen (*e.g.* anti-dsDNA), the name of the associated disease (*e.g.* anti-SS-A, as antigen A associated with Sjögren syndrome (SjS)) or the name of the first patient (*e.g.* anti-Sm as Smith). Within the ANA family, a group of physiological fluid-soluble macromolecules that can be extracted from the nucleus is covered by the term extractable nuclear antigens (ENA). Six antigens that represent ENA are SS-A (Ro60), SS-B (La), Sm, RNP, Scl-70, and Jo-1. Except nuclear this term refers also to cytoplasmic proteins, therefore the nomenclature is not entirely correct. Also, in the light of the constant broadening of the spectrum of clinically relevant autoantibodies, this term became obsolete but is still in wide use by clinicians (*1*).

In the presence of positive ANA, it is advisable to test for specific autoantibodies within the ANA family that are known to be related to certain CTDs in terms of clinical diagnosis, subsyndrome categorization, prognosis, or indication of the development of overlapping syndromes. Due to their presence years before the appearance of evident disease, these antibodies, can provide useful prognostic information regarding the clinical course or complications (*9*–*19*). Antinuclear antibodies specificities with these characteristics (but not limited to) are presented in Table 1.

**Table 1:** Clinically most relevant ANA specificities in inflammatory connective tissue diseases

**Autoantibody**	**Frequency of detection in different CTDs**	**Clinical significance**
anti-dsDNA	> 95% in active SLE with renal involvement 50–70% in active SLE without renal involvement < 40% in inactive SLE	ACR and SLICC classification criterion for SLE Prognostic marker for SLE (a marker of renal involvement, disease activity, use in therapy monitoring)
anti-SS-A (Ro60)	60–96% in primary SjS 40–60% in secondary SjS 25–60% SLE 60–100% SCLE 90% NLE	ACR/EULAR classification criterion for primary SjS Associated with extraglandular manifestations Found in mothers of children with NLE
anti-Ro52/TRIM21	17–63% SjS 23% SLE 20% SSc 30% of patients with the antisynthetase syndrome (in up to 72% of patients with positive anti-Jo1) in non-CTD diseases (28% PBC, 17% AIH)	Found in various autoimmune diseases
anti-SS-B (La)	40–70% in primary SjS 5–50% in secondary SjS 19–30% in SLE 25–80% in SCLE 70% NLE	Usually occur with SS-A antibodies Co-occurrence with SS-A antibodies usually correlates with fewer renal manifestations
anti-Sm	5–10% SLE	ACR and SLICC classification criterion for SLE High specificity for SLE
anti-RNP	100% MCTD, 13–32% SLE	Serological hallmark of MCTD (when present in high titer)
anti-Topo I/Scl70	65% in diffuse SSc	ACR/EULAR classification criterion for SSc Related to the more rapidly progressive systemic form of SSc
anti-CENP B	57–82% of patients with CREST syndrome 3–12% of patients with diffuse cutaneous Sc	ACR/EULAR classification criterion for SSc Associated with slowly developing limited cutaneous form of SSc (CREST syndrome)
anti-RNA-Pol III	3–19% in SSc	ACR/EULAR classification criterion for SSc Associated with diffuse skin manifestations and renal crisis
anti-Jo-1	24–30% IIM	ACR/EULAR classification criterion for adult and juvenile IIM Associated with interstitial pulmonary fibrosis
anti-PM/Scl	24–55% polymyositis/scleroderma overlap syndrome, 8–12% IIM, 1–16% SSc	Diagnostic marker for polymyositis/scleroderma overlap syndrome
anti-PCNA	3% SLE	Previously considered to be very specific for SLE
anti-ribosomal P	10–35% SLE	High specificity for SLE
anti-histones	92–95% drug-induced lupus, 50–80% SLE	High specificity for drug-induced lupus
anti-nucleosomes	56–90% SLE	High specificity for SLE
dsDNA – double stranded DNA. CTDs – Connective tissue diseases. SLE – Systemic lupus erythematosus. ACR – American College of Rheumatology. SLICC – Systemic Lupus International. Collaborating Clinics Classification Criteria for Systemic Lupus Erythematosus. SS-A (Ro60) – antigen A associated with Sjögren syndrome (60 kDa ribonucleoprotein). SjS – Sjögren syndrome. EULAR – European League Against Rheumatism. SCLE – Subacute cutaneous lupus erythematosus. NLE – Neonatal lupus erythematosus. Ro52/TRIM21 – 52 kDa ribonucleoprotein/ Tripartite motif-containing protein 21. SSc – Systemic sclerosis. PBC – Primary biliary cholangitis. AIH – Autoimmune hepatitis. CHB – Congenital heart block. SS-B (La) – antigen B associated with Sjögren syndrome. Sm – Smith antigen. RNP – ribonucleoprotein complex. MCTD – Mixed connective tissue disease. Scl-70/Topo-I – 70kDa antigen associated with scleroderma/Topoisomerase I. CENP-B – centromere protein B. CREST – Calcinosis, Raynaud’s syndrome, Esophageal dysmotility, Sclerodactyly, and Telangiectasia. RNA-Pol-III – RNA polymerase III. Jo-1 – histidyl-tRNA synthetase. IIM – Idiopathic inflammatory myopathies. PM/Scl – antigen associated with Polymyositis / Scleroderma overlap syndrome. PCNA –proliferating cell nuclear antigen. ribosomal P – ribosomal P protein.		

Recently, an increasing number of myositis-specific autoantibodies (MSAs) and myositis-associated autoantibodies (MAAs) have been detected in IIMs, which are useful for the subclassification of phenotypes, predicting prognosis, and determining the management. Consequently, numerous line immunoassays (LIA) became commercially available comprising different panels of MSAs and MAAs to achieve higher sensitivity since most of these autoantibodies are highly specific but present in up to 20% of patients (*20*–*22*).

The autoantibodies detected in serum samples of patients with autoimmune disease are high-avidity pathogenic autoantibodies of IgG isotypes (*10*). Although IgA and IgM can also be present, the association of these isotypes with CTD is less specific in comparison to IgG isotype (*9*).

## 1. Preanalytical issues

### Indications for ANA testing

Testing for ANA should be performed only in patients with symptoms of autoimmune rheumatic disease because weak ANA reactivity can be present in many non-rheumatic conditions (viral infection, paraneoplastic neurologic syndromes (PNS), liver disease, chronic fatigue syndrome, various cancers) and healthy individuals (in particular, pregnant women, women over 40 years, and elderly persons) (*10*).

The gold standard method for ANA detection is indirect immunofluorescence (IIF) using HEp-2 cells (human laryngeal epidermoid carcinoma cell line type 2) substrate and is referred to as the unique ANA-screen assay. Its definition as the gold standard is primarily based on the high sensitivity for SLE, despite the limited specificity, and a role in classification criteria for various CTDs (*1*, *23*).

It has been shown that ANA without any clinical significance may be found in 30% of healthy subjects at a titre of 1:40, and in 5% at a titre of 1:160 (*9*). Antinuclear antibodies screen assay shows high diagnostic sensitivity for certain CTDs (SLE (90-95%), primary Sjögren Syndrome (75%), scleroderma (85-90%), and MCTD (100%)), but it has relatively low specificity (*9*). Accordingly, patient pre-selection is very important to reduce the number of false-positive results and detection of autoantibodies out of a logical clinical context.

Recommendations for ANA determination indications:

1. Testing for ANA is recommended only in patients with symptoms related to CTD, in suspicion of AIH, and follow-up of patients with JIA.
2. The ANA-screen assay should be used for diagnostic purposes only and not for follow up of disease activity or therapy response.

### Sample type and stability

The specimen of choice is serum. It can be stored at 4 °C for two to three days and for a longer period it should be stored at - 70 °C (*24*). Storage at - 20 °C is generally acceptable for up to six months. Repeated freezing and thawing cycles may cause denaturation of immunoglobulins and should be avoided. Numerous manufacturers of commercial tests for detection of ANA, ENA, or anti-dsDNA recommend equally serum and plasma as the acceptable sample. There are no specific demands regarding transport conditions while respecting the above-mentioned stability requirements.

Only the standard patient preparation for routine laboratory analysis needs to be followed and, according to available data, the sampling time is not influenced by therapy.

Most manufacturers declare that highly haemolytic, icteric, and lipaemic samples should not be used, however, without specifying interfering concentrations. Therefore, if the manufacturer suggests avoiding haemolytic, icteric, or lipaemic samples, specific concentrations of interfering substances should be provided within package inserts or on-demand. Otherwise, it is advisable that each laboratory perform interference testing for the method in use.

Recommendation for the sample type:

1. Serum is the recommended sample type for the detection of autoantibodies.

### Quality control assessment

Special concern should be applied to quality control (QC) issues. Regarding the type of control material, minimal requirements should follow the manufacturer recommendation that usually addresses the performance of QC with manufacturer-provided positive and negative control samples. In the case of line blot or western blot methods, internal control (iQC) of the complete procedure is usually provided with the “control” line within each strip complemented with the lot specific QC sample usually applicable once per test kit. However, the use of manufacturer-independent control samples is highly encouraged. For the quantitative tests, a proper QC sample should be at the level close to the cut-off that is rarely met with the manufacturer recommended QC samples. Therefore, a native patient sample previously confirmed to be positive or negative for a specific antibody could be a good alternative. Furthermore, desired near cut-off or clinical decision level can be achieved by dilution of a positive patient sample with the negative one. The use of previously determined negative and positive patient sera as an intra-laboratory control sample is a more sensitive tool for the detection of lot-to-lot variation that can have a direct impact on clinical decision especially in the case of anti-dsDNA level used for monitor disease activity (*25*).

Potential disadvantages of a native patient sample use as an internal QC sample are instability and quantity issues. In contrast to commercial QC samples, native patient samples are not stabilized, and therefore stability under recommended storage conditions over time should be validated before use as a QC sample. Sodium azide (100-300 µg/mL) can be used as the preservative for this purpose (*24*). Before the implementation of the intra-laboratory control sample, the acceptance criteria of the result must be established. It can be defined simply in terms of qualitative concordance with previous results or in terms of acceptable quantitative deviation.

In terms of the IIF method, the use of a native patient sample as QC allows at the same time the control of the repeatability of the ANA IIF pattern and the titre. Use of the QC sample with a predefined titre is also recommended for the IIF method. It should be noted that the detectable differences of ANA IIF method are typical + or - 2 serial, twofold dilutions (*24*) and are recommended as criteria for acceptance of difference between two measurements of the control sample. For solid-based assays, the acceptable difference between successive measurements of the control sample is defined by the inter-assay coefficient of variation (CV %) of the method, for example, for the enzyme-linked immunosorbent assay (ELISA) method it is + or - 2 standard deviations (SD) which approximate ± 30% to 40% based on the intra-assay CV of 15% to 20% (*24*).

Use of the borderline positive QC, whether commercial or native patient sample, assures the check of the sensitivity of both microscope and the observer (*25*).

Another issue is the frequency of iQC. Internal control should be performed with every new reagent lot, irrespective of the method and frequency of patient samples testing. Regarding the same lot of reagent, ideally, iQC samples (positive/negative/borderline titre control) should be included in every batch of manually prepared slides for ANA determination with the IIF method. The same goes for solid-based assays in the case of measurement performed in batches while in the case of random automated methods performed on a daily basis the control procedure should be performed once per day, preferably at the start of the measuring procedure. However, for laboratories with low–to-medium sized throughput, this scheme is unlikely to be economically feasible. In line with it, the laboratory can optimize the frequency of iQC procedure by performing the analysis of retrospective data of iQC (in a respectable time frame, at least 6 months) and estimation of the sigma quality level to prove the stability of the analytical process. The sigma quality level provides information on the frequency of the occurrence of the various defects (in this case, the number of iQC results outside the defined acceptable criteria/total number of iQC results for individual parameters) (*26*). Once the frequency of the iQC has been established, the error rate needs to be regularly reviewed (at least annually) to verify the stability of the process and in the case of the observed decrease of the sigma quality level to increase the frequency of iQC. Another level of intra-analytical phase control is monitoring the proportion of negative results in the total number of the particular tests performed (both, ANA screen and specific antibodies). To achieve a proper insight into the performance of ANA testing within the laboratory, participation in an external quality assurance (EQA) scheme is mandatory.

Recommendations for quality control assurance:

1. Minimal requirements for quality control sample type should follow the manufacturer’s recommendation.
2. Use of the patient sample previously confirmed to be positive or negative for a specific antibody as the iQC sample is highly recommended.
3. iQC assurance should be performed with every new reagent lot.
4. Minimal requirements for the frequency of the iQC assurance for the same lot of reagent:
  once per batch of patient samples for the measurements not performed on a daily basis
  once per day, at the start of the measurement procedure, for the random automated methods performed on a daily basis.
  An exception of minimal requirements for the frequency of iQC assurance can be made in the case of the acceptable sigma quality level estimated on data collected during a minimum 6 months period.
5. Participation in an EQA scheme is mandatory.

### Rational algorithm

The introduction of ANA reflex testing has been shown in clinical practice as a good way to improve the efficiency of laboratory diagnostic of CTD with shortening the time to diagnosis while saving resources (*27*). Rational algorithms proposed in the literature mostly suggest the “gold standard” method, IIF on HEp-2 cells, as the most optimal screening test. Accordingly, the choice of an optimal ANA reflex test should be guided by the IIF pattern and titre as well as clinical indication. If alternative screening immunoassay is used, like solid-phase assay based on a finite set of nuclear antigens, then the results of such test should be reported with a disclaimer reporting limits of such testing.

**Figure 1:**
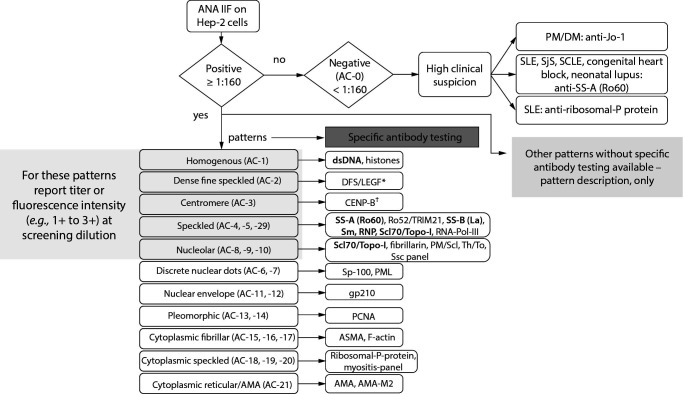
Algorithm of ANA IIF positive samples specific antibody testing depending on fluorescence pattern. Each pattern is designated with a corresponding alphanumerical code (see the following section for explanation). Minimally available specific antibody tests are designated with bold letters. *DFS70/LEGF reflex testing only if no ENA are confirmed; ^†^CENP-B antibody testing is not obligatory since fluorescence pattern is highly specific. ANA – antinuclear antibodies. IIF – indirect immunofluorescence. HEp-2 – human laryngeal epidermoid carcinoma cell line type 2. PM/DM – polymyositis/dermatomyositis. Jo-1 – histidyl-tRNA synthetase. SLE – systemic lupus erythematosus. SjS – Sjögren syndrome. SCLE – subacute lupus erythematosus. SS-A (Ro60) – antigen A associated with Sjögren syndrome (60 kDa ribonucleoprotein). dsDNA – double-stranded DNA. DFS70/LEGF – 70 kDa antigen associated with dense fine speckled fluorescence pattern on HEp-2 cells/ lens epithelium-derived growth factor. CENP-B – centromere protein B. Ro52/TRIM21 – 52 kDa ribonucleoprotein/ Tripartite motif-containing protein 21. SS-B (La) – antigen B associated with Sjögren syndrome. Sm – Smith antigen. RNP – ribonucleoprotein complex. Scl-70/Topo-I – 70kDa antigen associated with scleroderma/Topoisomerase I. RNA-Pol-III – RNA polymerase III. PM/Scl – polymyositis/scleroderma associated antigen. Th/To – nucleolar 7–2/8–2 RNA-protein complex. SSc – Systemic sclerosis. Sp100 – soluble nuclear protein. PML proteins – Promyelocytic Leukemia proteins. gp-210 – nucleoporin 210. PCNA – proliferating cell nuclear antigen. ASMA – anti-smooth muscle antibodies. AMA – antimitochondrial antibodies. M2 – E2 subunit of pyruvate dehydrogenase complex. ENA – extractable nuclear antigens.

Positive IIF test is intended to be followed by antigen-specific immunoassay, depending on fluorescence ANA pattern (Figure 1). It is advisable to focus on those specific ANA that are known to be clinically important (*10*, *28*). The specific antibody testing should minimally involve the solid-based assays which include classical ENA antigens (SS-A (Ro60), SS-B (La), Sm, RNP, Scl-70, and Jo-1) and dsDNA (*6*, *28*–*31*). Multiplex bead assays (addressable laser bead immunoassay, ALBIA) allow the determination of different ANA specificities simultaneously (usually dsDNA, ENA, CENP B).

To further improve rationalization, assays with a mixture of nuclear autoantigens coupled to a solid matrix (solid-based screen assays) can precede individual tests which can then be neglected in the case of the negative result of the screen assay. These assays are available in the form of ENA-screen, comprising only classical ENA, or as a connective tissue disease (CTD) screen which comprises a wider spectrum of clinically important autoantigens (*32*).

In case of a negative IIF test, selected specific antibody testing should nevertheless be carried out in the context of strong clinical suspicion on certain diagnoses (for example, anti-Jo-1 in the case of PM/DM, anti-SS-A (Ro60) in case of SjS, congenital heart block or neonatal lupus, anti-ribosomal P protein in the case of SLE) (*32*–*34*).

In case of clinical suspicion for distinct ANA-associated rheumatic diseases such as idiopathic inflammatory myopathies (IIM) and systemic sclerosis (SSc), it is advisable to perform immunoassays for disease-specific autoantibody profiles which are commercially available (*35*).

Detection of antibodies targeting 70 kDa antigen associated with dense fine speckled fluorescence pattern on HEp-2 cells (anti-DFS70) is recommended due to the added value in exclusion of autoimmune disease diagnosis (but only if no ENA is recognized) due to its negative association with SARD (*36*).

Since in real-life requests are usually not accompanied by clinical information, recently it has been suggested to combine IIF with solid-based screen assay to gain maximum sensitivity and specificity for CTD diagnosis (*32*, *37*). The efficiency of such a strategy seems to be disease associated with the best efficiency observed for SLE and SjS while for SSc no added value was obtained in comparison to the algorithm with IIF as the first-line test and solid-based assay performed on IIF positive samples (*38*).

### Recommendations for rational algorithm:

1. The recommended first-line test in the detection of ANA is the IIF screen test on HEp-2 cells.
2. Testing for specific ANA should be performed only in the cases of positive ANA IIF screen test with titre ≥ 1:160, guided by the fluorescence pattern. Exceptions are related to the aforementioned clinical indications (*i.e*. ant-SS-A or Jo-1 due to the low sensitivity of the IIF method for these antibodies).
3. If the complete evaluation of ANA (screen and specificity confirmation) was not included in the request, it is highly advisable to perform a complete evaluation or should be advised in the comment of the report, as a minimal request.

## 2. Analytical issues

### ANA screen assay

It is well known that for tests with screening purposes high sensitivity is mandatory, whereas for confirmatory tests the high specificity is primary. Antinuclear antibodies determination by IIF on HEp-2 cells or HEp2000 (transfected HEp-2 cells with SS-A cDNA) as a recommended substrate, possesses all the characteristics to be employed as a first-line screening test in the diagnosis of CTD and is therefore considered as the “gold“ standard technique and a reference method for ANA screening. Fluorochrome (fluorescein isothiocyanate, FITC) – labeled anti-human-Ig conjugate used in the ANA IIF test should be IgG specific (polyvalent conjugates may also be used but they can detect increased percentages of clinically insignificant antibodies) (*1*, *2*, *23*, *32*, *39*). Using the IIF on HEp-2 for ANA screening, more than 100 different autoantibodies targets can be detected, far more than any other commercially available solid based assay (*2*, *40*).

In 2015 The International consensus on antinuclear antibody pattern (ICAP) defined and described three major groups of staining patterns: nuclear, cytoplasmic, and mitotic, that can be seen by IIF on HEp-2 cells. Each pattern is designated with a corresponding alphanumerical code (AC). Patterns within each group have been described in detail on the official website https://www.anapatterns.org/.

**Figure 2:**
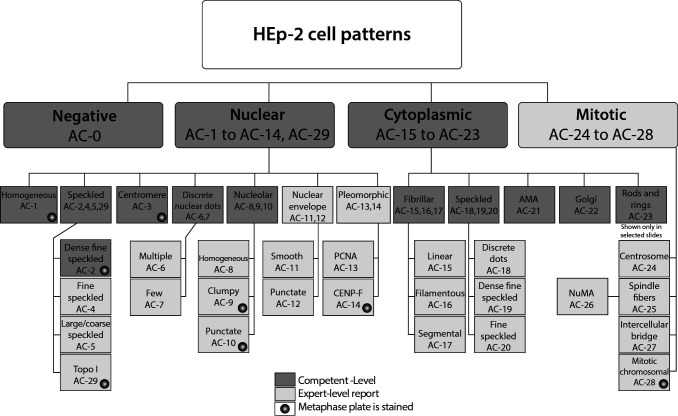
ICAP Nomenclature and classification tree of ANA IIF patterns on HEp–2 cell substrate (https://www.anapatterns.org/). ICAP – The International consensus on antinuclear antibody pattern. HEp – 2 – human laryngeal epidermoid carcinoma cell line type 2. ANA – antinuclear antibodies. IIF – indirect immunofluorescence. PCNA – proliferating cell nuclear antigen. CENP-F – centromere protein F. AMA – antimitochondrial antibodies.

By ICAP recommendation ANA screen performed by IIF should be reported as positive in cases of positive nuclear but also in cases of clear cytoplasmic and mitotic immunofluorescence patterns (*2*, *41*). The ICAP nomenclature and classification of ANA IIF patterns on HEp-2 cells is shown in Figure 2.

The ICAP intention/recommendation for pattern recognition is to differentiate them on two levels:

Competent-level: patterns that are easily recognizable and are strongly recommended for reporting. That also includes patterns whose clinical relevance still isn’t clear/defined.

Expert-level: patterns that are more challenging to recognize and which can be recognized/reported only by observers that have expert-level experience.

Like in other screening tests, when defining the cut-off titre it is important to ensure the appropriate sensitivity and specificity of the test on defined dilution. It is well known that a positive ANA can be found not just in patients with autoimmune disease but also in healthy individuals. At a lower cut-off titre the sensitivity of the test is increasing but the specificity of the test, as a tool for diagnosis of CTD is lower and with a higher rate of positive results in healthy individuals. Few studies confirmed the use of cut-off titre at 1:160 as optimal for differentiation of healthy individuals from those with CTD (*42*–*44*). The International recommendations for the assessment of ANA also recommend setting up a screening cut off for ANA IIF on HEp-2 cells at 1:160 for the adult population, but it also emphasizes that a negative result at cut off of 1:160 does not mean the complete exclusion of disease (*1*). No recommendation/consensus is made for the cut-off titre when ANA screen is performed in children population (< 16 years) (*45*, *46*).

According to the International recommendations for the assessment of ANA, a sample found to be positive on ANA IIF screening should be further diluted in twofold dilutions up to the highest dilution (titre) to which the fluorescence can be seen as a result of autoantibody reactivity (*1*). Our recommendation is that titration should be performed at least up to the clinically significant titre of 1:640 or up to 1:5120 maximally since titre above the latter does not have added clinical value (*32*). Reporting of ANA titre is important for differentiation of healthy individuals from patients with CTDs since there is an established preponderance of low titre reactivity among the ANA positive healthy individuals compared to patients with CTD (*42*). Along with the titre, a report on a positive ANA result should be accompanied with a description of fluorescence pattern according to ICAP AC-nomenclature (Table B in the Appendix) since distinctive pattern profiles have been associated with CTD but also with ANA positive healthy individuals. Some patterns, such as nuclear homogenous (AC-1), coarse speckled (AC-5), and centromere (AC-3) IIF patterns are highly associated with disease-restricted autoantibodies and are observed almost exclusively in samples of patients with CTD or individuals with high risk for CTD development. In contrast, nuclear dense fine speckled IIF pattern (AC-2) when present without a concurrent pattern, and usually in high titre, is almost exclusively observed in apparently healthy individuals and patients with diverse non-CTD inflammatory conditions (*37*). It should be kept in mind that despite the high correlation with specific autoantibodies, observed IIF patterns are used only as a guide to specific tests (exception is centromere pattern), either as a further step in the algorithm (Figure 1) or simply as a recommendation on the report.

Although considered as the gold standard method for the detection of ANA, IIF assay has numerous disadvantages; it is prone to human bias, is labor-intensive, time-consuming, and needs experience. It is important to note the low sensitivity of ANA IIF for some specific autoantibodies: SS-A, Ro52, ribosomal-P protein, Jo-1, and other myositis-specific autoantibodies (*37*). Also, components of the microscope such as light power or lens magnification as well as methods used for HEp-2 cell preparation substantially contribute to the variability of the assay. Some of these disadvantages have been reduced with the introduction of automated microscopic systems based on digital acquisition and analysis of IIF images by pattern recognition algorithms, but not all ANA patterns can be recognized with these systems (*47*, *48*).

The term “ANA screen” should be used exclusively for the IIF method since it is the only method that comprises all nuclear antigens, though some of them in a low level of expression (*1*). Solid-based assays, often used as an alternative to IIF, represents the mixture of defined autoantigens (native or recombinant) coupled to different solid supports, known under the term CTD screen assays. These assays cannot be considered as ANA screen due to a restricted number of autoantigens. If the assay comprises only antigens covered by the term ENA, this assay is called ENA screen assay. Novel technologies enabled the identification of numerous new autoantigens in CTD. Widening the spectrum of included autoantigens improved sensitivity of the new generation of solid-based assays making them almost an IIF complementary tool for ANA detection. It is important to note that these assays cannot be the alternative to the IIF assay when ANA testing is requested as part of the autoimmune hepatitis work up. Most commonly, solid-based immunoassays used as screening methods include standard ELISA which is increasingly being replaced with novel technologies such as fluorescence enzyme immunoassay (FEIA) and more recent, chemiluminescent immunoassay (CLIA). The advantages of solid-based assays are based on high analytic specificity and sensitivity, better reproducibility, less labor-intensive and time-consuming, not subjective, and do not require training and expertise like IIF, all of which ensures reliability and consistency (*36*, *47*). Solid-based screen assays are essentially qualitative and should be interpreted as such irrespective of the quantitative measurement in the background (ratio).

The major drawbacks of these methods are the use of a limited number of purified or recombinant autoantigens, lack of standardization, and the prevalence of “false negative” ANA results, although some of them with questionable clinical significance. A recent meta-analysis compared IIF with solid-based immunoassays used as an initial screening method (*49*). No significant difference between ELISA and IIF (cut-off 1:80) was found both in terms of sensitivity and specificity. The sensitivity of CLIA was also comparable to IIF while for FEIA it was significantly lower. On the other hand, the specificities of both CLIA and FEIA were higher than IIF. According to these data, the combination of IIF (most sensitive) and CLIA or FEIA (most specific) should achieve the highest diagnostic accuracy. However, despite the benefits of using new automated technologies in the diagnosis of ANA-associated autoimmune rheumatic diseases (AARD), the common view of the world’s leading organizations (ACR, EASI, WHO, International Union of Immunological Societies (IUIS)) is that the IIF is the reference method for ANA screen (*47*, *48*, *50*).

### ANA specific tests (ENA including)

Nowadays, many technologies are available for determining ANA (including ENA) specific tests, and they differ according to technology, as well as the ability to provide quantitative results. The most commonly used methods are (in alphabetical order): addressable laser bead immunoassay (ALBIA), CLIA, ELISA, FEIA, and line immunoassay (LIA). Depending on the technology, determination of ANA specificity can be performed by individual assay or in the form of an assay which provides determination of several specificities at the same time (ELISA, LIA, and ALBIA). Simultaneous determination of defined, usually clinically most relevant ANA specificities saves time for diagnostic work out. ANA (ENA) specific assays can be qualitative (LIA with the possibility of semi-quantitative interpretation by scanning the lines), semi-quantitative or quantitative, although quantification is justified only for anti-RNP due to the clear association with specific clinical entities (MCTD).

### Antibodies to dsDNA

Antibodies to double-stranded DNA are highly specific for the diagnosis of SLE (*51*). Apart from diagnostic, these antibodies have also prognostic usefulness since the dynamic of titre is in direct proportion to disease activity. A rise in titre often precedes SLE exacerbation by several weeks. High titres have been associated with lupus nephritis (*11*, *52*). Even though there is an international standard available, different methods give different concentrations of anti-dsDNA for the same samples. These differences could be attributed to the molecular properties of used dsDNA antigens, as well as the experimental conditions of the assay. Also, the complexity of the antigen elicits a highly diverse immunologic response. Considering the stated, it is very important to monitor patients at the same laboratory using the same method (*53*, *54*).

The major difference between anti-dsDNA antibody subpopulations, regarding clinical significance, is antibody avidity. In contrast to low avidity antibodies, those with high avidity proved to be specific for SLE, more closely associated with renal involvement and related to disease activity (*55*, *56*).

Detection of both high and low avidity anti-dsDNA in assays such as ELISA results in a lower specificity for SLE than *e.g.* an immunofluorescence test for antibodies to native DNA (nDNA), using the kinetoplast of *Crithidia luciliae* as the substrate (CLIFT) or the CLIA (*57*).

Due to the high specificity and positive predictive value for the SLE, CLIFT is used for confirmation of a positive result obtained with a less specific method. The low diagnostic sensitivity of the CLIFT assay (20-35%), limits its utility in the SLE case finding (*56*). Before choosing an anti-dsDNA assay, the testing environment (primary or secondary/tertiary care) should be taken into consideration. In a secondary/tertiary care environment, where primarily clinical immunologist or rheumatologist orders the test, and the pre-test probability is high, a high sensitivity assay is preferred (*52*).

### Interferences

Immunoassays are well known to be prone to interferences due to the complexity of antigen-antibody interaction and low concentration of an analyte. Potential interferences include non-specific pre-analytical aspects (lipaemia, haemolysis) but what is more important and more challenging for detection, a significant number of analyte-dependent interferences (*58*). In assays used in humoral immunodiagnostics of autoimmune diseases, the autoantibody is the analyte of interest (“antigen“ in the context of antigen-antibody reaction) while the method can employ one antibody (detection) or two antibodies (capture and detection). These assays are vulnerable to interferences from endogenous antibodies as heterophile antibodies (HA), autoantibodies, and human anti-animal antibodies (HAAA). Among interfering autoantibodies, RF is of particular interest since it is present in the majority of CTD patients (for example, in up to 80% of SjS patients) so the information provided by the manufacturer regarding the interfering cut-off for RF is of utmost importance. With the growing use of novel therapies with monoclonal antibodies, the impact of the interference of HAAA on immunoassays for autoantibody detection gained importance. The next interference to be thought of is the antigen excess (in this case autoantibody excess), the effect known as the high dose hook effect. Besides the solid-based assays, this effect can also occur in commonly used IIF assays such as ANA screen assay (*59*). One should suspect the interference in the case of discrepancies of the test result with clinical condition or discrepancies between the two assays measuring the same analyte. The example for the last is the first line combined testing with ANA IIF and ENA/CTD screen assay which yield negative ANA IIF result and positive result of the ENA/CTD screen further confirmed with positive individual ANA specificity as a second-line test. In this case, antigen excess interference in the IIF test should be excluded with further serial dilutions of the sample. Due to its wider measuring range in comparison to other methods, this type of interference is unlikely in CLIA methods for ANA detection. It is essential for laboratory specialists to be aware of the limitations of the method in use as well as the vulnerability to potential interferences.

Recommendations for analytical issues:

1. The reference method for the detection of ANA is IIF on HEp-2 cells (or HEp2000) substrate.
2. Antinuclear antibodies screen dilution can be adopted from the manufacturer recommendation (usually 1:80 or 1:100) or be user-defined provided it corresponds to the 95th percentile of healthy controls.
3. Titration of positive ANA IIF is recommended at least to a clinically significant titre of 1:640.
4. Anti-dsDNA has to be determined with quantitative assay and use of CLIFT assay is optional, solely as a confirmation of avidity of positive anti-dsDNA antibodies determined with the quantitative assay.
5. In the context of the use of solid-based screen assays, specific confirmatory tests for antigens included in screen assay should be performed only in the case of a positive result, without exceptions.
6. Quantification of individual ENA specificities is mandatory only for RNP antibodies since the presence of these antibodies at a high level is the hallmark of MCTD.
7. Interference in the assay, whether IIF or solid-based, should be suspected in the case of discrepancy with a clinical condition or between the results of two assays measuring the same analyte. The investigation of possible interferences should include serial dilution of the sample or repeat analysis with another method.

## 3. Postanalytical issues

Reporting of the result is equally important as the result itself. In the report several key points require particular attention: a) nomenclature; b) specification of antigens included in the case of solid-based screen assays; c) units; d) cut-off; e) method in use, and f) comment.

### Nomenclature

One of the first steps in harmonizing the laboratory reports between laboratories is the use of unified nomenclature so that any confusion or misinterpretation by the clinician can be safely avoided. Recommended nomenclature is given in Table A and B in the Appendix and should be applied in the report of the result.

### Specification of antigens

In the case of solid-based screen assays, included antigens need to be stated in the report. An example is the ENA-screen assay that *in sensu strictu* encompasses 6 antigens: SS-A, SS-B, Sm, RNP, Scl-70 (Topo-1), and Jo-1. Nowadays, it is well known that the distinction of SS-A antigens as Ro60 and Ro52 (TRIM21) is clinically relevant since specific reactivity is associated with different clinical scenarios (*1*). In accordance with it, the report must contain information concerning the distinction between SS-A (Ro60) and Ro52 antibodies. Another example is Sm antibodies which react with nine different polypeptides, but mostly with BB’ and D polypeptides. Recent data confirmed that only SmD (particularly SmD_3_ subpopulation) is considered the most SLE-specific antigen while tests that include BB’ antigens fail to differentiate patients with SLE from those with other autoimmune diseases. As in the case of anti-SS-A antibodies, specification of target protein within the same antibody family gives additional information regarding the test specificity and explains the origin of result discrepancies between two different methods for Sm antibodies (*60*, *61*).

This issue is particularly concerned with tests based on different mixtures of defined nuclear and cytoplasmic antigens that are commonly used instead of ANA IIF test. As mentioned before, this test cannot be considered equal to ANA IIF. Since clinicians need to be aware that the negative result of such a test does not necessarily mean a negative ANA but refers only to negative antibodies that target antigens included in the mixture, these antigens should be clearly stated in the report.

### Units of measurement

It is recommended that the results of the ANA IIF screen assay are reported as a titre and not simply as positive or negative (*1*, *28*).

However, one should bear in mind that variation in ANA titre over time has no established clinical significance and should not be used as guidance for therapy adjustment (*62*). Generally, antibodies as a measurand belong (as most of the proteins) to the group of analytes which are not well-defined chemical entities traceable to the International System (SI) units but are rather heterogeneous in human samples. Therefore, the reference material, in this case, represents only a surrogate for the analyte measured in the patient sample and the result cannot be expressed in SI units but rather in terms of arbitrary units for example WHO international units (IU/mL) (*63*, *64*). Within the ANA family, the reference standard is available only for anti-dsDNA antibodies, and therefore it is the only ANA specific antibody which results should be reported in IU/ml.

The results for quantitative as well as for semi-quantitative assays for other ANA specificities (other than anti-dsDNA) should be given in arbitrary units, AU/mL or relative units, RU/mL, or chemiluminescence units (CU) since there is no available reference material for these specificities. However, there is still no solid evidence that quantification of specific ANA antibodies other than anti-dsDNA and anti-RNP (also nucleosomes although not commonly included in routine practice) has added value in diagnostic workup or disease surveillance.

### Cut-off level

Screening dilutions in use for ANA IIF assay on HEp-2 cells varies between manufacturers with 1:80 and 1:100 being the most common. Laboratories often interpret screening titre as the cut-off titre or use the most frequently recommended 1:160 titre as the cut-off to increase the specificity of the test for autoimmune diseases (*65*, *66*).

The optimal cut-off is highly dependent on a screened population and differs between primary and secondary care. For example, for general practitioners, the high negative predictive value (NPV) of ANA is of utmost interest so that exclusion of systemic rheumatic disease can be done with great certainty (*67*, *68*).

The capture antigen quality differs among manufacturers so that the cut-off values of solid-based assays greatly varies from one manufacturer to another, both for screen and confirmatory assays. Even the applied cut-off for anti-dsDNA assays which are calibrated against the same standard vary widely (from 15 IU/ml to > 100 IU/ml). This is primarily due to the high heterogeneity of dsDNA antibodies among individual patients but also can be attributed to the aforementioned variability of capture antigen (*69*).

Usually, the cut-off recommended by the manufacturer is used but verification on the local population is strongly encouraged. While planning the cut-off verification, one should keep in mind the intended use of the assay in a routine setting. High sensitivity is mandatory for a screening assay to minimize the number of missed patients in contrast to confirmatory tests that should have high specificity (*40*). Verification of the cut-off value should be performed according to CLSI guidelines for semi/quantitative or qualitative tests depending on the performance of the test, and it is recommended to use age and sex-matched sera (*70*, *71*).

### Methods in use

Within the report of the result of ANA testing, the laboratory should specify each method used. Clinicians have to be aware of potential discrepancies between results for the same patient using different methods.

### Comments

The report of the results of ANA testing should be accompanied with corresponding comments wherever it may improve diagnostic workup. This primarily concerns suggestions to the clinician for follow up testing based on the result of the ANA IIF assay. For example, in the case of the homogenous fluorescence of nucleoplasm with positive chromatin plate of metaphase cells, anti-dsDNA testing should be suggested but limited to clinical suspicion of SLE. In the case of speckled fluorescence, ENA testing should be advised. Another example is finding coarse granular filamentous cytoplasmic staining that matches the pattern of antibodies other than ANA, antimitochondrial antibodies (AMA), in which case the clinician should be suggested for further testing. Another aspect of improvement with added comments is the interpretation of results that might help a clinician in matching the puzzles of diagnosis. For example, a positive dense fine speckled pattern as a result of ANA IIF test followed with confirmed single reactivity to DFS70 antigen indicates that the presence of the systemic autoimmune rheumatic disease is unlikely (*40*, *72*, *73*)

Also, the distinction between SS-A (Ro60) and Ro52 has been available only recently so that many clinicians are not familiar with the clinical significance of Ro52 and interpretation of the positive result might be helpful.

### Repeated ANA measurements

Determination of ANA is a primarily a diagnostic test and serial monitoring of ANA titre is not indicated because changes in ANA titre do not correlate with disease activity and cannot be used for estimating the efficiency of therapy (*1*, *62*). In the case of negative or low positive ANA, it is useful to repeat measurement only in patients with persistent or worsening clinical signs of CTD (*9*, *74*). Repeated requests for autoantibody determination represent a significant, unnecessary cost (*75*, *76*). Repeat requests for initially positive ANA in patients with clinically defined CTD are unnecessary unless there is a strong suspicion of disease phenotype change or appearance of another autoimmune rheumatic disease (*9*).

Seroconversion of the ENA, whether positive or negative, occurs infrequently. A recent study performed among SLE patients confirmed this frequency to be < 5% (*77*). The high-cost burden together with the lack of evidence that the initial result is prone to changes suggests that repeating ENA tests is unnecessary. In addition, the correlation of ENA level with disease activity has not been confirmed (*78*). Exceptions to the rule are again previously ENA negative patients with persistent or worsening clinical signs which indicate the evolution of CTD or previously positive ENA patients with clinical signs that indicate the appearance of another autoimmune rheumatic disease. Also, repeat determination of ENA is indicated as a part of preconception assessment of SLE patients because a positive SS-A and/or SS-B and Ro52 antibodies would prompt fetal echocardiography in search for congenital heart block (*79*).

Anti-dsDNA concentration is known to correlate with the disease activity and predicts the flares of SLE, although this relationship can be individual and seems to be highly dependent on the method for anti-dsDNA detection (relates to high avidity antibodies!) (*52*). Monitoring of disease activity in SLE is commonly accomplished using the SLE Disease Activity Index (SLEDAI), which includes anti-dsDNA and complement components (*64*). In line with this, repeat measurement of anti-dsDNA is clinically justified and the frequency of retesting is tailored according to the disease activity (*1*, *52*, *62*).

Recommendations for postanalytical issues:

1. IIF pattern should be reported along with titre according to recommended terminology given in Table B of the Appendix, along with the corresponding AC-number. Link to the ICAP website should be given in the comment of the report. A competent level of pattern recognition is the minimal requirement for reporting.
2. Solid-based screen assays that use a limited mixture of defined antigens should not be named as ANA screen test. Instead, the term given in Table A of the Appendix should be used together with the included antigens specified.
3. Results of solid-based screen assays that use a restricted mixture of defined antigens should be reported exclusively as qualitative.
4. Specification of the method used for ANA screen or screen on the restricted mixture of defined ANA antigens should be part of the report.
5. Specification of methods used for ANA specific antibodies (including ENA and dsDNA) should be part of the report.
6. Results of quantitative tests for ANA specific antibodies should be reported as follows:
  anti-dsDNA in IU/mL
  all other specificities in AU/mL or RU/mL or CU for CIA assays.
7. Repeat determination of ANA is justified:
  in initially negative or low titre positive patients, only in the case of persistent or worsening clinical symptoms.
  in the patient with clinically defined CTD, only if there is a change in the clinical manifestations that raises the suspicion of a change in the underlying disease or the appearance of associated rheumatic disease (overlap syndrome).
8. Repeat determination of positive dsDNA antibodies should be performed with the same quantitative method and in recommended intervals:
  6-12 months for inactive disease
  2-4 months in SLE patients with renal involvement, according to the estimation of disease activity (*62*).
9. Repeat determination of ENA:
  as a part of preconception assessment in SLE patients due to the reevaluation of previously negative SS-A (Ro60), Ro52, and SS-B (La) antibodies which are associated with NLE and its most severe complication, congestive heart block.
  if there is a change in the clinical picture that can be related to changing of disease phenotype or occurrence of associated rheumatic disease.

## Conclusion

The survey conducted in 2016 among Croatian laboratories performing diagnostic of autoimmune diseases shed a light on the huge diversity in all steps of the laboratory procedure for ANA testing. The reason partly lies in the different technologies in use but also in the lack of documented guidance for the preanalytical, analytical, and postanalytical phases of ANA testing. These recommendations have a goal to harmonize ANA testing on the national level concerning different technologies in use. One of the most valuable expected results of their application is the lack of confusion for clinicians produced by different algorithms and different reports for the same test.

## Appendix

**Table A:** Nomenclature on the report

Screen tests^a^
1. Antinuclear antibodies (ANA) – screen test^b^
2. Screen test for the restricted number of specific antinuclear antibodies (ANA): specify included antigens^c^
3. ENA screen test: specify included antigens^d^
Tests for specific antibodies^a^
1. Antibodies to double-stranded DNA (anti-dsDNA)
2. Antibodies to SS-A (Ro) antigen (anti-SS-A (Ro))^e^
3. Antibodies to SS-A (Ro60) antigen (anti-SS-A (Ro60))
4. Antibodies to Ro52 antigen (anti-Ro52)
5. Antibodies to SS-B (La) antigen (anti-SS-B (La))
6. Antibodies to Smith antigen (anti-Sm)^e^
7. Antibodies to RNP antigen (anti-RNP)^f^
8. Antibodies to Topoisomerase I (anti-Topo I/Scl70)
9. Antibodies to Jo-1 antigen (anti-Jo-1)
10. Antibodies to centromere protein B (anti-CENP B)
11. Antibodies to PM/Scl antigen (anti-PM/Scl)
12. Antibodies to proliferating cell nuclear antigen (anti-PCNA)
13. Antibodies to ribosomal P protein (anti-ribosomal P)
14. Antibodies to histones
15. Antibodies to nucleosomes
16. Antibodies to RNA-polymerase III (anti-RNA-Pol III)
17. Antibodies to DFS70 antigen (anti-DFS70)
^a^Method should be specified either along with the test name or in the legend of the report. ^b^Refers exclusively on IIF test on HEp-2 or HEp-2000 substrate. ^c^Refers to solid-based screen tests commercially available under the common name CTD-screen. ^d^ENA screen test refers exclusively on test comprising following antigens: SS-A (Ro) (when there is no distinction between Ro52 and Ro 60) or SS-A (Ro60) and Ro52 (TRIM21), separately; SS-B (La); Sm (or SmD according to manufacturer specification); RNP (U1-RNP or Sm/RNP, according to manufacturer specification); Scl-70 (Topo-1) and Jo-1. In the case that the screen test includes additional antigens that are not included in ENA term, use the nomenclature given under number 2. ^e^SS-A (Ro) – if there is no distinction between Ro52 and Ro60; ^e^ Alternatively, anti-SmD in the case that manufacturer declares specificity exclusively for SmD antigen. ^f^anti-RNP term comprises both anti-U1-RNP and anti-Sm/RNP. IIF – indirect immunofluorescence. HEp-2 – human laryngeal epidermoid carcinoma cell line type 2 substrate. CTD – connective tissue diseases. ENA – extractable nuclear antigens. RNP – ribonucleoprotein complex.

**Table B:** ICAP nomenclature for the description of fluorescence patterns and corresponding clinical significance

**ICAP code**	**Fluorescence pattern**	**Clinical significance***
	**Nuclear**	
**AC-1**	Nuclear homogenous fluorescence	SLE, drug-induced lupus, JIA
**AC-2**	Nuclear dense fine speckled	Rare in SjS, SSc, and SLE, more often in healthy individuals and different non-autoimmune inflammatory diseases (atopic dermatitis, asthma, *etc.*)
**ICAP code**	**Fluorescence pattern**	**Clinical significance***
**AC-3**	Centromere fluorescence	Limited cutaneous SSc, PBC
**AC-4**	Nuclear fine speckled fluorescence	SjS, SLE, dermatomyositis
**AC-5**	Nuclear large/coarse speckled	MCTD, SLE, SSc
**AC-6**	Fluorescence of multiple nuclear dots	PBC, SARDs, dermatomyositis
**AC-7**	Fluorescence of few nuclear dots	SjS, SLE, SSc, polymyositis, asymptomatic individuals
**AC-8**	Homogenous nucleolar fluorescence	SSc, SSc/PM overlap syndrome
**AC-9**	Clumpy nucleolar fluorescence	SSc
**AC-10**	Punctate nucleolar fluorescence	SSc, SjS
**AC-11**	Smooth nuclear envelope fluorescence	SLE, SjS, seronegative arthritis
**AC-12**	Punctate nuclear envelope fluorescence	PBC
**AC-13**	PCNA-like fluorescence	SLE, other disorders
**AC-14**	CENP-F – like fluorescence	Carcinomas, other disorders
**AC-29**	Topo-I like fluorescence	SSc
	**Cytoplasmic**	
**AC-15**	Cytoplasmic fibrillar linear fluorescence	MCTD, chronic active hepatitis, cirrhosis, myasthenia gravis, Morbus Crohn, PBC, long-term haemodialysis, rare in SARDs
**AC-16**	Cytoplasmic fibrillar filamentous fluorescence	Infective or inflammatory disorders, long-term haemodialysis, alcoholic liver disease, SARDs, psoriasis, healthy individuals
**AC-17**	Cytoplasmic fibrillar segmental fluorescence	Myasthenia gravis, Morbus Crohn, ulcerative colitis
**AC-18**	Fluorescence of cytoplasmic discrete dots/GW body-like	PBC, SARDs, different neurologic and autoimmune disorders
**AC-19**	Cytoplasmic dense fine speckled fluorescence	Anti-synthetase syndrome, polymyositis/dermatomyositis, SLE, juvenile SLE, neuropsychiatry SLE
**AC-20**	Cytoplasmic speckled fluorescence	Anti-synthetase syndrome, polymyositis/dermatomyositis, limited SSc, idiopathic pleural effusion
**AC-21**	Cytoplasmic reticular fluorescence/AMA	Often in PBC and SSc, rare in other SARDs
**AC-22**	Cytoplasmic polar/Golgi like fluorescence	Rare in SjS, SLE, RA, MCTD, GPA, idiopathic cerebral ataxia, paraneoplastic cerebral degeneration, virus infections
**AC-23**	“Rods & Rings “ fluorescence	HCV patients after IFN-γ/ribavirin therapy, rare in SLE, Hashimoto thyroiditis and healthy individuals
	**Mitotic**	
**AC-24**	Centrosome fluorescence	Rare in SSc, Raynaud syndrome, infection with viruses and mycoplasmas
**AC-25**	Fluorescence of spindle fiber	Rare in SjS, SLE, and other CTDs
**AC-26**	NuMA-like fluorescence	SjS, SLE, other disorders
**AC-27**	Fluorescence of intercellular bridge	Rare in SSc, Raynaud syndrome and malignancies
**AC-28**	Mitotic chromosomal fluorescence	Rare in discoid lupus erythematosus, chronic lymphatic leukemia, SjS, and polymyalgia rheumatica
*Report should contain description of fluorescence pattern with corresponding AC number, without clinical significance. Instead, link to the ICAP web site where corresponding clinical significance can be found should be given in the comment of the report. ICAP – International consensus on antinuclear antibody pattern. SLE – Systemic lupus erythematosus. JIA – juvenile idiopathic arthritis. SjS – Sjögren syndrome. SSc – Systemic sclerosis. PBC – Primary biliary cholangitis. MCTD – mixed connective tissue disease. SARD – systemic autoimmune rheumatic diseases. PCNA – Antibodies to proliferating cell nuclear antigen. CENP-F – centromere protein F. Scl-70 – 70kDa antigen associated with scleroderma. PM – polymyositis. GW body – G (glycine) and W (tryptophan) containing body. AMA – antimitochondrial antibodies. HCV – hepatitis C virus. GPA – granulomatosis with polyangiitis. IFN-γ – interferon gamma. CTD – connective tissue diseases. NuMA – nuclear mitotic apparatus protein.		
