# Corrigendum to: Assessment of antinuclear antibodies (ANA): National recommendations on behalf of the Croatian society of medical biochemistry and laboratory medicine

**DOI:** 10.11613/BM.2022.011201

**Published:** 2022-02-15

**Authors:** Andrea Tešija Kuna, Lovorka Đerek, Vedrana Drvar, Ana Kozmar, Katarina Gugo

**Affiliations:** 1 Department of Clinical Chemistry, Sestre Milosrdnice University Hospital Center, Zagreb, Croatia; 2 Clinical Department of Laboratory Diagnostics, University Hospital Dubrava, Zagreb, Croatia; 3 Clinical Department of Laboratory Diagnostics, Clinical Hospital Center Rijeka, Rijeka, Croatia; 4 Department of Laboratory Diagnostics, University Hospital Center Zagreb, Zagreb, Croatia; 5 Department of Medical Laboratory Diagnostics, University Hospital Center Split, Split, Croatia

This is a correction of Biochem Med (Zagreb) 2021;31(2):020502, DOI: https://doi.org/10.11613/BM.2021.020502.

Since the publication of the article, the authors have noticed some errors:

1. On page 2, under the heading Introduction, third paragraph, the last sentence should be: “Antinuclear antibodies represent the classification criteria of most CTD, while it is a one of the fundamental serological markers for diagnosis of autoimmune hepatitis type I (AIH type I) as an organ-specific autoimmune disease and validated risk factor for the development of uveitis in patients with juvenile idiopathic arthritis (JIA) (7,8).” instead of “Antinuclear antibodies represent classification criteria of most CTD while it is a fundamental parameter for diagnosis of autoimmune hepatitis (AIH) as an organ-specific autoimmune disease and validated risk factor for the development of uveitis in patients with juvenile idiopathic arthritis (JIA) (7,8).”
2. On page 4, under the heading Sample type and stability, the recommendation should state: “Serum is the recommended sample type for the detection of ANA autoantibodies.” Instead of “Serum is the recommended sample type for the detection of autoantibodies.”
3. On page 5, under the heading Quality control assessment, last paragraph, a reference is added to the penultimate sentence: “Another level of intra-analytical phase control is monitoring the proportion of negative results in the total number of the particular tests performed (both, ANA screen and specific antibodies) (*27*)”. The new reference is listed below.
4. On page 6, under the heading Rational algorithm, second paragraph, the last sentence should be: “Multiplex tests on microparticles such as the immune method with laser addressable microparticles (Addressable laser bead immunoassay, ALBIA), or Luminex method, allow the determination of different ANA-specific antibodies simultaneously (usually dsDNA, ENA, CENP B).” instead of “Multiplex bead assays (addressable laser bead immunoassay, ALBIA) allow the determination of different ANA specificities simultaneously (usually dsDNA, ENA, CENP B).”

The authors apologize for any inconvenience caused to the readers.
